# Dirac Signature in Germanene on Semiconducting Substrate

**DOI:** 10.1002/advs.201800207

**Published:** 2018-05-04

**Authors:** Jincheng Zhuang, Chen Liu, Zhiyong Zhou, Gilberto Casillas, Haifeng Feng, Xun Xu, Jiaou Wang, Weichang Hao, Xiaolin Wang, Shi Xue Dou, Zhenpeng Hu, Yi Du

**Affiliations:** ^1^ Institute for Superconducting and Electronic Materials (ISEM) Australian Institute for Innovative Materials (AIIM) University of Wollongong Innovation Campus North Wollongong NSW 2500 Australia; ^2^ BUAA‐UOW Joint Centre Beihang University Haidian District Beijing 100091 P. R. China; ^3^ Beijing Synchrotron Radiation Facility Institute of High Energy Physics Chinese Academy of Sciences Beijing 100049 P. R. China; ^4^ School of Physics Nankai University Tianjin 300071 P. R. China; ^5^ Electron Microscopy Centre University of Wollongong Wollongong NSW 2525 Australia; ^6^ Center of Materials Physics and Chemistry, and Department of Physics Beihang University Beijing 100191 P. R. China; ^7^ School of Physics Beihang University Haidian District Beijing 100091 P. R. China

**Keywords:** 2D materials, Dirac fermions, field‐effect transistors, germanene, scanning tunneling microscopy, transmission electron microscopy

## Abstract

2D Dirac materials supported by nonmetallic substrates are of particular interest due to their significance for the realization of the quantum spin Hall effect and their application in field‐effect transistors. Here, monolayer germanene is successfully fabricated on semiconducting germanium film with the support of a Ag(111) substrate. Its linear‐like energy–momentum dispersion and large Fermi velocity are derived from the pronounced quasiparticle interference patterns in a √3 × √3 superstructure. In addition to Dirac fermion characteristics, the theoretical simulations reveal that the energy gap opens at the Brillouin zone center of the √3 × √3 restructured germanene, which is evoked by the symmetry‐breaking perturbation potential. These results demonstrate that the germanium nanosheets with √3 × √3 germanene can be an ideal platform for fundamental research and for the realization of high‐speed and low‐energy‐consumption field‐effect transistors.

## 1 Introduction

The Dirac equation dictates that the propagation of a 2D electron gas (2DEG) in a honeycomb periodic potential results in electronic Dirac fermion systems, in which electrons behave as massless quasiparticles with high mobility due to their linear energy–momentum dispersion. For example, graphene, silicene, and germanene are typical 2D electronic Dirac materials because the dynamics of their low‐energy electrons, modulated by two equivalent atomic sublattices, fulfills such requirements.^1^, ^2^, ^3^, ^4^, ^5^, ^6^ These materials show exceptionally high Fermi velocities and are promising for realizing the quantum Hall effect, Klein paradox, and nontrivial quantum states.^7^ Among them, germanene is theoretically predicted to have a larger energy gap due to its greater spin–orbital coupling strength.^2^, ^3^ This is of benefit for the realization of electronic devices such as field‐effect transistors, which require controlling and even switching off the electrical conductivity by means of gate electrodes. Unfortunately, free‐standing (FS) germanene featured by the sp^2^ configuration has not been observed in nature because intrinsic Ge—Ge bonds only take energy favored sp^3^ hybridization. Recently, 2D germanene with a low‐buckled honeycomb atomic arrangement has been stabilized by the support of metallic substrates, including Au(111),^8^, ^9^, ^10^ Pt(111),^11^ Al(111),^12^ Cu(111),^13^ and Sb(111),^14^ forming abundant superstructures given raise by different germanene–substrate interactions. These substrate‐induced superstructures break the lattice symmetry and induce the superlattice potentials to modulate the electronic properties. Nevertheless, the electronic states derived from p_z_ orbitals of Ge atoms, which give the π electrons near the Dirac points with the almost linear energy dispersion, are strongly hybridized with the metallic substrate states. As a result, wave functions derived from the p_z_ orbitals are delocalized into the metallic substrate. This may lead to the absence of Dirac fermion characteristics.^15^ Furthermore, the application of germanene in electronic functional devices requires exfoliation of germanene from the metallic substrate or deposition on nonmetallic substrates to eliminate the possible current bypass effect. The strong germanene–metallic–substrate interaction significantly increases the difficulty in adopting the former method. Thus, the realization of germanene with Dirac fermion characteristics on semiconducting or insulating substrates is crucial, not only for fundamental research but also for potential applications in nanotechnology.

In this work, we successfully fabricated monolayer germanene with a (√3 × √3)*R*30° supercell on a semiconducting Ge(111) surface with the support of a Ag(111) substrate, which is verified by both scanning tunneling microscopy (STM) and scanning transmission electron microscopy (STEM). A linear‐like energy–momentum band dispersion relation has been deduced from the quasiparticle interference (QPI) patterns, regardless of the thickness of the underlying Ge(111) film. Furthermore, the supercell provides the reciprocal lattice vector connecting the two inequivalent Dirac cones in germanene, which breaks the chiral symmetry through the interaction between different valley states as well as splits the Dirac cone in the Brillion zone center by different on‐site potentials. Our work provides a feasible way to fabricate a Dirac electronic material with an energy gap, which paves the way to the development of high‐performance electronics.

## 2 Results and Discussion

**Figure 1:**
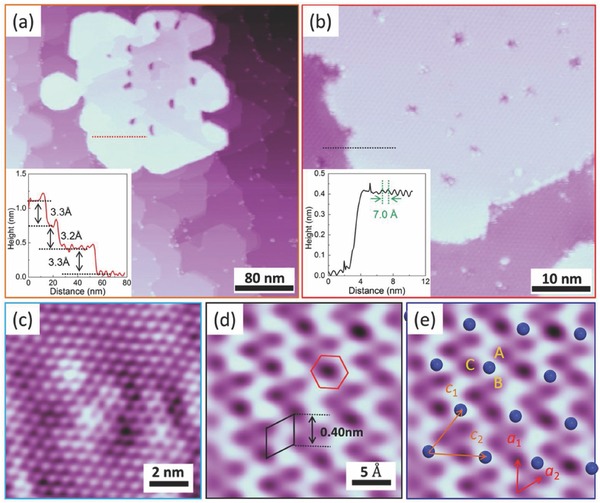
STM images of thick germanium nanosheets. a) Large‐scale STM image of germanium nanosheets on Ag(111) substrate (*V*_bias_ = 2 V, *I* = 50 pA). Inset is the line profile along the red dashed line. b) Enlarged view of STM image of germanium nanosheets (*V*_bias_ = –2 V, *I* = 50 pA). Inset is the line profile along the black dashed line. c) High‐resolution STM image of the surface structure of germanium nanosheets (*V*_bias_ = 1 V, *I* = 50 pA). d) Atomic resolution STM image explored by a small bias voltage and large tunneling current. The black solid rhombus stands for the unit cell of 1 × 1 germanene (*V*_bias_ = 1 mV, *I* = 4 nA). e) STM image from panel (c) with blue balls labeled for the top Ge atoms.

The growth dynamics and surface reconstructions of germanium nanosheets were studied in detail by STM, as shown in **Figure** **1** and in the Supporting Information. It was found that the initially deposited Ge atoms insert themselves into the Ag(111) surface and form a Ag_2_Ge surface alloy (see Figure S1, Supporting Information), which is consistent with the previous reports.^16^, ^17^ The Ag_2_Ge structure could be driven into the disordered honeycomb arrangement by additional Ge deposition atoms with surface adatoms assembling themselves in the forms of dots, dimers, trimers, tetramers, and hexamers (see Figure S2, Supporting Information). Thus, the additional Ge atoms “pull out” the Ge atoms in Ag_2_Ge alloy to form the disordered structure due to the stronger strength of Ge—Ge covalent than that of Ge—Ag interfacial bonds. Further deposition leads to the formation of germanium nanosheets, as shown in Figure 1a. Flat terraces are formed on the surfaces of germanium nanosheets (Figure 1a) with terrace height of 3.25 ± 0.05 Å (inset of Figure 1a). The enlarged STM images (Figure 1b,c) demonstrate the close‐packed hexagonal arrangement of surface protrusions with a periodicity of 7.0 Å. When a small sample bias of 1 mV and a large tunneling current (4 nA) were applied during the scanning process, a honeycomb arrangement with low‐buckled atomic structure was revealed (Figure 1d). The surface periodicity given by the distance between the nearest two dark depressions, labeled by the black solid rhombus, is around 4.0 Å, which is in a good agreement with the lattice constant of the simulated FS germanene (3.97–4.06 Å).^2^, ^18^ Thus, the arrangement of buckled‐up atoms, marked by blue balls in Figure 1e, corresponds to the √3 × √3 germanene superstructure on the surface. The atomic‐resolution STM image in Figure 1d results from the addition of signals from the buckled‐down atoms, which is induced by the small bias voltage and large tunneling current.

**Figure 2:**
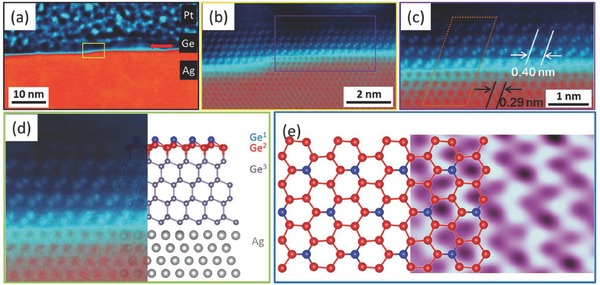
Cross‐sectional structure of germanium nanosheets. a) Wide view, HAADF STEM image of germanium nanosheets with the protective Pt capping layer and Ag(111) substrate indicated. b) High‐resolution HAADF STEM image of the region marked by the yellow solid frame in panel (a). c) Enlarged view of the area of the purple solid frame in panel (b). The orange frame is plotted to represent the commensurate superlattice between Ge atoms and Ag atoms. d) Schematic diagram of the evolution from the relaxed model of the cross‐sectional atomic structure to the experimental HAADF STEM image (from right to left). e) Schematic diagram of the evolution from the relaxed model of the atomic surface structure to the experimental STM image (from left to right). Ge1, Ge2, and Ge3 stand for the buckled‐up Ge atoms (blue balls) of the √3 × √3 superstructure, buckled‐down Ge atoms (red balls) of the √3 × √3 superstructure, and Ge atoms (purple balls) of bulk Ge(111).

Because STM only reveals the surface topography, it is necessary to further explore the ordered stacking structure of the germanium nanosheets. We used aberration‐corrected STEM (AC‐STEM), combined with the focused ion beam technique, to investigate the cross‐sectional structure. The image acquired with the high‐angle annular dark field (HAADF) detector (**Figure** **2**a) is sensitive to the atomic number *Z* (contrast ≈*Z* ^3/2^) and shows unambiguous contrast between the Ag(111) substrate, deposited germanium nanosheets, and amorphous Pt capping layer. The germanium nanosheets exist in the form of islands, consistent with the STM results (see Figure S3, Supporting Information) and indicating a Volmer–Weber growth mode. The germanium nanosheets grow over the substrate step edges, suggesting a structure that is distinct from the Ag(111) substrate. The high‐resolution image (Figure 2b) implies an abrupt atomic interface between the germanium nanosheets and the Ag(111) substrate, without any miscible area containing both Ge and Ag, indicating higher chemical interaction between Ge atoms than the Ag–Ge interaction. The lattice constant of the deposited germanium nanosheets is around 4.0 Å (Figure 2c), where the 3 × 3 superlattice (4.0 Å × 3 = 12.0 Å) matches well with the 4 × 4 unit cell of the Ag(111) substrate (2.9 Å × 4 = 11.6 Å). It should be noted that the value of the lateral lattice constant of bulk Ge(111) is 4.0 Å, and thus, the high‐resolution HAADF results (Figure 2c) exhibit classic bulk Ge in a diamond cubic crystal structure with <111> being the dominant growth direction (Figure 2d). This is also supported by our in situ Raman measurements (see Figure S5, Supporting Information), where the Raman spectrum of germanium nanosheets is almost identical to that of Ge(111) single crystal. Based on the above results, we propose the structural mode of the germanium nanosheets as follows: a diamond‐structured Ge(111) thin film with a surface of √3 × √3 superstructure germanene (with respect to 1 × 1 FS germanene) is formed on Ag(111) substrate, where the lattice constant of the 3 × 3 unit cell of Ge(111) matches that of the 4 × 4 unit cell of Ag(111). The evolutions from the cross‐sectional structural mode to the HAADF image (Figure 2d), and from the surface structural mode to the high‐resolution STM image (Figure 2e), indicate the agreement between the structural model and the experimental results.

**Figure 3:**
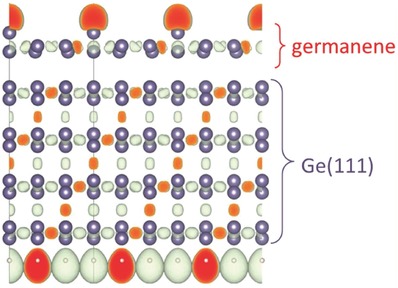
Side view of ELF iso‐surface taken at a value of 0.86 of the cross‐sections between germanium pairs.

The interactions between the Ge atoms on the top layer and the interactions between the top‐layer Ge atoms and beneath Ge atoms in bulk Ge(111) are investigated by the calculation of the electron localization function (ELF), as it gives information about the nature of the interactions between atoms via electron localization. **Figure** **3** displays the side view of ELF between germanium pairs, which gives clear evidence that the interaction between topmost germanene layer and underlying four‐layer Ge(111) is weaker than that between two Ge atoms in √3 × √3 germanene. It is also weaker than the interlayer interactions in the Ge(111) substrate. The results show a moderate interaction between topmost layer and underlying substrate, which is comparable to that between the well‐recognized 2D materials and their various substrates (see Figure S6 and Table S1, Supporting Information), and indicates 2D nature of √3 × √3 germanene.

**Figure 4:**
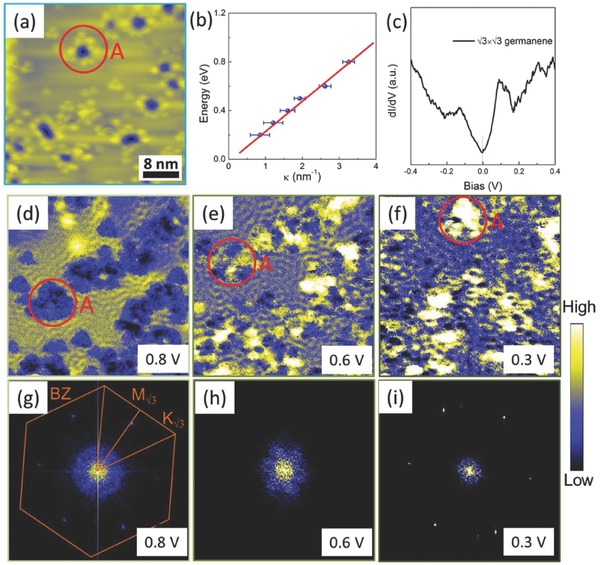
QPI of √3 × √3 superstructure. a) Topography of √3 × √3 supercell with point defects (*V*_bias_ = −0.5 V, *I* = 300 pA). b) Energy–momentum dispersion of √3 × √3 superstructure derived from the FFT patterns of the STS maps at varied bias voltages. The red solid line is plotted to show a linear fit to the data. c) *dI*/*dV* curves of √3 × √3 germanene taken at 77 K. The position of the DP is labeled by red dashed line. d–f) STS maps of the same area as panel (a), collected with the bias at 0.8, 0.6, and 0.3 V, respectively. The red solid circles and “A” denote the position of one of the point defects. g–i) *k*‐space maps obtained by the FFT at different bias voltages of 0.8, 0.6, and 0.3 V in panels (d), (e), and (f), respectively. The hexagon stands for the Brillouin zone (BZ) of the √3 × √3 superstructure, and *M*_√3_ and *K*_√3_ label the *M* point and *K* point in the BZ of the √3 × √3 superstructure, respectively.

In order to investigate the electronic structure of germanene, we performed STS measurements (*dI*/*dV* curves and maps). A typical *dI*/*dV* curve taken at 77 K is shown in **Figure** **4**c, where a V‐like shape locating at around Fermi level is observed. This feature is similar to that in graphene, which is attributed to the characteristic of 2D Dirac system.^19^ The scanning tunneling spectroscopy (STS) mapping was performed in a surface region with point defects (Figure 4a), which could act as scattering centers to induce the pronounced local density of states (LDOS) oscillation. The QPI patterns were identified, in which its wavelengths are modulated by the bias voltage (Figure 4d–f). Similar Friedel oscillations with varied wavelength could also be observed near the step edges on the germanene surface (see Figure S7, Supporting Information). It is notable that the LDOS oscillation is a result of quasiparticle scattering among different points of the constant energy contour (CEC). Thus, we can deduce the energy–momentum dispersion by plotting the *E*(κ) curve, where κ is the radius of the CEC at the *Γ* point with 2κ = |*q*|, where *q* is the intravalley scattering wave vector determined from radius of the fast Fourier transform (FFT) patterns of the STS maps. The linear‐like energy–momentum dispersion was explored (Figure 4e) with the Fermi velocity around (3.7 ± 0.3) × 10^5^ m s^−1^ and is regarded as one dispersion branch of the Dirac cone. It indicates the existence of Dirac fermions in this material. Furthermore, the intercept value for κ = 0 gives the Dirac energy, which is close to that of Dirac point position (dip position labeled by red dashed line in Figure 4c). The STS mapping of the bare Ag(111) substrate was also performed to eliminate the effects of the 2DEG from the Shockley surface state.^20^ The FFT images exhibit a circular shape (see Figure S8, Supporting Information), regardless of the bias voltage, and they clearly deviate from the hexagonal case in the √3 × √3 superstructure.

**Figure 5:**
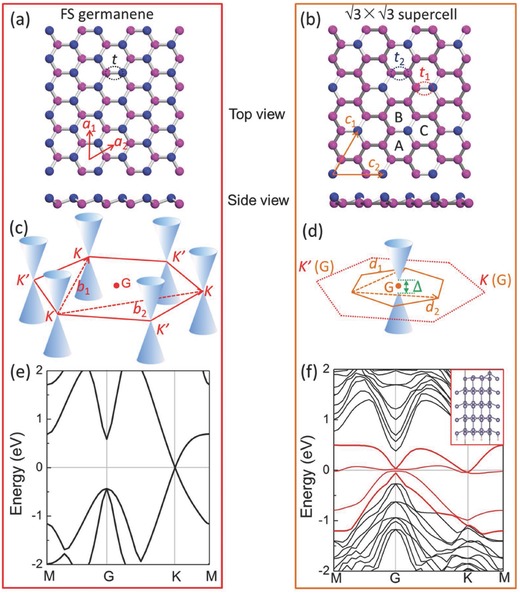
DFT simulations of both FS germanene and the √3 × √3 superstructure on the surface of Ge(111) nanosheets. a,b) Schematic diagrams of the lattice structure of FS germanene and the √3 × √3 superstructure from both top views and side views, respectively. (**a**_1_, **a**_2_) and (**c**_1_, **c**_2_) represent the lattice vectors of FS germanene and the √3 × √3 restructured germanene, respectively. In FS germanene, all the hopping amplitudes have the same value *t*. Two types of Ge—Ge bonds in the √3 × √3 supercell, denoted as *t*_1_ and *t*_2_, are distinguished due to the presence of one buckled‐up atom in the unit cell (six Ge atoms). c,d) Schematic diagrams of the first Brillouin zone of FS germanene and the √3 × √3 supercell, respectively. (**b**_1_, **b**_2_) and (**d**_1_, **d**_2_) represent the reciprocal‐lattice vectors of FS germanene and the √3 × √3 supercell, respectively. “∆” is the label for the energy gap opening after the formation of the √3 × √3 supercell. e) Simulated band structure of FS germanene. f) Projected electronic structure of the √3 × √3 superstructure on the surface of a four‐layer‐thick Ge(111) nanosheet.

In fact, the CEC of the Dirac cone at the *K* or *K*′ points for 2D elemental materials, such as graphene and low‐buckled FS silicene/germanene, is isotropic and circular close to the Dirac point (DP), but it becomes trigonal at positions far away from the DP due to the interactions with the three nearest conical bands.^2^, ^21^ The FFT of the STS map in *k* space (Figure 4g–i), however, exhibits a hexagonal rather than a circular or trigonal shape, which could have the following explanation. The lattice vectors used to portray the √3 × √3 superstructure (**c**_1_, **c**_2_) are √3 times that of 1 × 1 FS germanene (**a**_1_, **a**_2_) with a 30° rotation (**Figure** **5**a,b). Thus, reciprocal‐lattice vectors of this superstructure (**d**_1_, **d**_2_) couple the *K* and *K*′ points of the Brillouin zone (BZ) of 1 × 1 phase, causing the Dirac cones located at the *K* and *K*′ points of the BZ of 1 × 1 phase to be folded into the center *Γ* point of the BZ of the √3 × √3 superstructure (Figure 5d). The two sets of trigonal cones at the *K* and *K*′ points with antiparallel directions could form a hexagonal‐shaped cone at the *Γ* point when the energy level is far away from the DP. The hexagonal CEC of the Dirac cone gives rise to the hexagonal scattering vector in the FFT images of STS maps, as the backscattering is the primary process because of the enhanced phase space.^22^, ^23^ On the other hand, the backscattering should be strongly attenuated by the states with antiparallel pseudospins, which will give fast‐decaying oscillations and result in absence of QPI patterns in the energy region near the Dirac point.^24^ The hexagonal‐wrapped CEC combined with the possible density of states shift could provide additional, unprotected, nesting, or near nesting scattering vectors (with non‐antiparallel pseudospins) to enhance the pronounced LDOS oscillations, leading to the observation of QPI patterns.^23^, ^24^ Moreover, the oscillations cease below Fermi level, which is due to the possible hybridization between Dirac band in germanene and bulk valence band in bulk Ge(111) as the energy bandgap of germanium, is only around 0.65 eV.^25^ That is why we observed QPI patterns only at limited energy range above Fermi energy.

In addition to the band folding effect, the √3 × √3 superstructure also periodically alters the nearest‐neighbor hopping amplitudes with two values, where *t*_1_ is the hopping parameter for one‐third of the bonds bridging between one buckled‐up Ge atom and one buckled‐down Ge atom, and *t*_2_ is the parameter for the remaining two‐thirds of the bonds connecting two buckled‐down Ge atoms (Figure 5b), which forms the so‐called Kekulé construction.^26^ This periodical spatial modulation of the hopping parameters in its Hamilton equation gives rise to the background, yielding a chiral mixing and leading to the gap between the two species of Dirac cone in graphene.^27^ Another order parameter contributing to the energy gap size in the Dirac spectrum is the staggered chemical potential, taking on varied values in different sublattices of the honeycomb lattice.^28^ In our √3 × √3 superstructure, this perturbation is correlated with the on‐site energy potential of different Ge atoms in the surface. The band structure of √3 × √3 germanene on the surface of a four‐layer‐thick Ge(111) nanosheet was calculated (see Figure 5f and Figure S9, Supporting Information), where an energy gap with a value up to 78 meV is opened at Dirac cones (red curves). The value of the computed energy gap promises the usability of this material at room temperature. The preservation of the √3 × √3 surface reconstruction, regardless of the thickness of the underlying Ge(111) nanosheets, provides flexibility for modulating the dielectric properties of the semiconducting Ge(111) nanosheets layer by layer. It should be noted that the minimum thickness of germanium nanosheets is around 1.4 ± 0.1 nm, which is much less than the value of thickness (≈5–10 nm) in the current semiconductor manufacturing. All of these results promise the development of germanene‐based electronics.

In order to obtain deep insight into the factors responsible for the band dispersion near the Fermi surface, the numerical results from a tight‐binding (TB) model with different order parameters were obtained. The Hamiltonian of the superstructure could be written as

(1) $$H\;=\sum_{i\;\;=\;\;1}^{6}\varepsilon_{i}a_{i}^{+}\;a_{i}-\left(\sum_{i\;=\;1}^{6}\sum_{j<\;\;i}t_{ij}a_{i}^{+}a_{j}+h.c.\right)$$

where ε stands for the on‐site energy of Ge atoms. When the differences in on‐site energies are ignored, there will be four degenerate roots with zero values, leading to the absence of an energy gap at the *Γ* point of the Brillouin zone of this superstructure (Note 10, Supporting Information). In fact, the on‐site energies are different in the superstructure from our density functional theory (DFT) calculations. The hopping parameter *t*_2_ is set to 1.30 eV, which is also determined by the DFT calculations. The *t*_1_ is tuned to see how the band structure changes with the coupling strength. After considering the variation of on‐site energies of different Ge atoms, the four bands degenerate and the energy gap opens at *Γ* point of BZ (see Figure S10c, Supporting Information). The two bands close to zero energy become more and more flat with the decrease in the value of *t*_1_ (see Figure S10, Supporting Information). The band structure around the *Γ* point is similar to the DFT results (Figure 5f) with *t*_1_ = 0.30 eV. The numerical results agree well with the physical picture that forming the superstructure results in decreasing orbital overlapping and increasing orbital energy for the buckled‐up Ge atoms, which means a smaller *t*_1_ and larger on‐site energy discrepancy in the numerical model, respectively. Although the TB model is a little rough, due to the fact that the included orbitals are not as many as in the DFT calculations, it clearly reveals the role of the order parameters in the superstructure, where the energy gap is mainly evoked by the discrepancy between the staggered chemical potentials, and the coupling between the buckled‐up atom and other atoms dominates the dispersion of the energy bands close to the Fermi level.

We have noticed the debate on the existence of √3 × √3 multilayer silicene on Ag(111).^29^, ^30^ A lot of works attributed the √3 × √3 multilayer silicene to the silicon (111) islands covered by a monolayer of Ag atoms, which is the well‐known Si(111)√3 × √3‐Ag reconstruction.^31^ This opinion is based on their similar surface structures observed in STM image and low‐energy electron diffraction intensity,^32^, ^33^, ^34^, ^35^ and transmission electron microscopy observations (TEM) for Si(111) patterns,^33^ and comparatively study of their electronic structures by STS and metastable atom electron spectroscopy.^29^, ^36^ On the other hand, the existence of √3 × √3 silicene is supported by Dirac cones in ARPES measurements,^5^, ^6^, ^37^, ^38^ QPI in STS results,^4^, ^23^, ^39^, ^40^ and 1 × 1 atomic structure and Moiré pattern induced by rotation between adjacent layers.^41^ Furthermore, a quasi‐2D electron gas state with parabolic dispersion has been identified in Ge(111)(√3 × √3) Ag surface.^42^ Thus, it is essential to rule out of the possibility of Ge–Ag substitutional surface alloy case and to confirm formation of √3 × √3 germanene in our samples. We performed X‐ray photoemission spectroscopy (XPS) and STEM combined with energy‐dispersive X‐ray spectroscopy (EDX) measurements to investigate the surface chemical nature of our sample.

**Figure 6:**
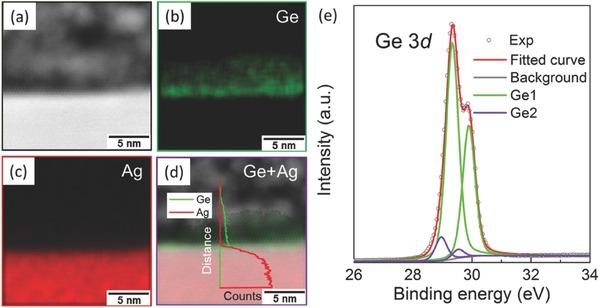
Surface chemical characterization. a) STEM image along with EDX element mapping for b) Ge, c) Ag, and d) Ge + Ag from the view of cross‐section. The line‐scan EDX spectra are superimposed into the panel (d) to show the dispersion of Ag and Ge. e) Representative Ge 3*d* core‐level XPS spectra of √3 × √3 germanene on Ge(111) with the support of Ag(111) taken at *hν* = 300 eV. The Ge1 and Ge2 peaks are attributed to Ge—Ge bonds in bulk Ge(111) and √3 × √3 germanene, respectively.

**Figure** **6** shows the STEM together with EDX elemental mapping images for both of Ag and Ge atoms from the view of the cross‐section. An abrupt interface between the germanium nanosheets and the Ag(111) substrate could be identified in Figure 2b–d, which is consistent with the high‐resolution STEM images in Figure 2b. More importantly, there is no any signal of Ag atoms in the Ge nanosheets, neither in the topmost germanene nor in the underlying Ge(111), as shown in Figure 6c. Moreover, the superimposed line‐scan of EDX spectra in Figure 6d implies that the concentration of Ag in Ge nanosheets is zero. It should be noted that surface atomic ratio between Si/Ge and Ag in the √3 × √3‐Ag mode is 1:1,^31^ which is high enough to guarantee the observation of Ag signal in the EDX mapping results. Thus, our EDX results show the direct and solid evidence for that there is no Ag atoms segregating into the topmost surface of our sample.

Figure 6e displays the Ge 3*d* core‐level XPS spectra of √3 × √3 germanene formed on Ge(111) with the support of Ag(111). The experimental data points are displayed with black dots, while the overall fitted curves are red line. The fitting results make it clear that there are two groups of bonding components, labeled as Ge1 and Ge2, respectively. The energy gap of the two peaks in each group is a constant value, indicating that the two fitting peaks in one group are related to two Ge 3*d*3/2 and 3*d*5/2 peaks, respectively. The Ge 3*d*5/2 spectrum appears as two peaks, 29.34 and 28.96 eV, both of which are close to that measured for germanene on Au(111)^8^ and germanene on Al(111).^43^, ^44^ Thus, the peaks in Ge1 group and Ge2 group are assigned to Ge—Ge bonds in bulk Ge(111) and Ge—Ge bonds in √3 × √3 germanene, respectively. Our XPS results indicate that there is no Ag atom in the surface forming Ag–Ge alloy, and hybridizations between Ge atoms are different in surface √3 × √3 germanene and underlying Ge(111). Moreover, the honeycomb‐chained triangle model and inequivalent triangle model were used to describe the atomic structure of the Si/Ge(111)‐√3 × √3 Ag surface, where each protrusion in STM images is contributed by one Ag trimer.^34^ However, these Ag trimmers do not exist on √3 × √3 germanene from atomic resolution STM image (Figure 1d), excluding the possibility of Ag‐terminated Ge(111) reconstruction.

## 3 Conclusions

In summary, we have demonstrated the formation of √3 × √3 germanene on Ge(111) nanosheets with the support of a Ag(111) substrate. A linear‐like energy–momentum dispersion is deduced from the QPI patterns under different energy levels, indicating the existence of Dirac fermions in the √3 × √3 restructured germanene. The DFT simulations exhibit a gap that has opened at the Dirac point due to the periodically altered hopping amplitudes and on‐site energies evoked by the Kekulé distortion. The high charge carrier mobility, a sizable energy gap corresponding to room temperature, and semiconducting Ge(111) nanosheets with tunable thickness acting as a dielectric layer present a possible avenue to realize nanotechnology applications of this material.

## 4 Experimental Section

*Synthesis of Germanium Nanosheets*: All samples used in this work were fabricated in an ultrahigh vacuum (<5 × 10^−11^Torr, UHV) preparation chamber equipped on STM. Clean Ag(111) substrates were prepared by argon ion sputtering and annealed at 750 K for several cycles. The germanium nanosheets were then deposited on the Ag(111) surfaces by evaporation of germanium from a heated germanium wafer. The deposition flux of Ge was 0.1 monolayers per minute (ML min^−1^). The Ag(111) substrate temperature was maintained at 450 K during the deposition process.

*Characterization of Structural and Electronic Properties*: The STM and Raman spectroscopy measurements were carried out by using a low‐temperature UHV STM/scanning near‐field optical microscopy system (SNOM 1400, Unisoku Co.) in UHV (<8 × 10^−11^Torr) at 77 K. The Raman spectra were acquired using a laser excitation of 532 nm (2.33 eV), delivered through a single‐mode optical fiber at 77 K in UHV. The spot size of the incident laser in the in situ Raman spectroscopy was about 3 µm in diameter. The differential conductance, *dI*/*dV*, spectra were acquired by using a standard lock‐in technique with a 10 mV modulation at 967 Hz. HAADF images and STEM spectra were obtained using a probe corrected JEOLARM‐200F operating at 200 kV with a Centurio energy dispersive spectroscopy solid‐state X‐ray detector. In situ XPS characterizations were performed at the Photoelectron Spectroscopy Station (Beamline 4B9B) in the Beijing Synchrotron Radiation Facility (BSRF) using a SCIENTA R4000 analyze, and variable photon energies were referenced to a fresh Au polycrystalline film.

*DFT Calculations*: All calculations were performed under the framework of DFT as implemented in the Vienna ab initio simulation package.^45^, ^46^ The Perdew–Burke–Ernzerhof functional was selected to describe the exchange–correlation interactions of the electrons.^47^ A 4.01 Å × 4.01 Å × 30.00 Å (α = β = 90°, γ = 120°) unit cell was used for the FS single‐layer model. A 6.93 Å × 6.93 Å × 30.00 Å (α = β = 90°, γ = 120°) unit cell was used for all the √3 × √3 models. A *Γ*‐center 13 × 13 × 1 K‐mesh sampling for the FS model and a *Γ*‐center 7 × 7 × 1 K‐mesh sampling for the √3 × √3 model with a 400 eV cut‐off energy on plane wave basis sets were used in the calculations. All models were full relaxed. The convergence criteria were 10^−5^eV for total energy and 0.02 eV Å^−1^ for the force on each atom.

## Conflict of Interest

The authors declare no conflict of interest.

## Supporting information

Supplementary
