# Supplementary material for: Dirac Signature in Germanene on Semiconducting Substrate
Source: Adv Sci (Weinh). 2018 May 4;5(7):1800207. doi: 10.1002/advs.201800207 (PMC6051399; doi:10.1002/advs.201800207)
Supplement: Supplementary file 1 — Supplementary [file ADVS-5-1800207-s001.pdf]

Copyright WILEY-VCH Verlag GmbH & Co. KGaA, 69469 Weinheim, Germany, 2018.

Supporting Information

for *Adv. Sci.,* DOI: 10.1002/advs.201800207

Dirac Signature in Germanene on Semiconducting Substrate

Jincheng Zhuang, Chen Liu, Zhiyong Zhou, Gilberto Casillas, Haifeng Feng, Xun Xu, Jiaou Wang, Weichang Hao, Xiaolin Wang, Shi Xue Dou, Zhenpeng Hu,* and Yi Du**

Supporting Information

Dirac signature in germanene on semiconducting substrate

Jincheng Zhuang, Chen Liu, Zhiyong Zhou, Gilberto Casillas, Haifeng Feng, Xun Xu, Jiaou Wang, Weichang Hao, Xiaolin Wang, Shi Xue Dou, Zhenpeng Hu,* and Yi Du**

Dr. J. C. Zhuang,[+] Mr. H. F. Feng, Dr. X. Xu, , Prof. X. L. Wang, Prof. S. X. Dou, Dr. Y. Du
Institute for Superconducting and Electronic Materials (ISEM), Australian Institute for Innovative Materials (AIIM), University of Wollongong, Innovation Campus, North Wollongong, New South Wales 2500, Australia
E-mail: yi_du@uow.edu.au

Dr. J. C. Zhuang, Mr. H. F. Feng, Prof. W. C. Hao, Prof. X. L. Wang, Prof. S. X. Dou, Dr. Y. Du
BUAA-UOW Joint Centre, Beihang University, Haidian District, Beijing 100091, China

Mr. C. Liu,[+] A/Prof. J. O. Wang
Beijing Synchrotron Radiation Facility, Institute of High Energy Physics, Chinese Academy of Sciences, Beijing 100049, China
E-mail: wangjo@mail.ihep.ac.cn

Mr. Z. Y. Zhou,[+] A/Prof. Z. P. Hu
School of Physics, Nankai University, Tianjin, 300071, China
E-mail: zphu@nankai.edu.cn

Prof. W. C. Hao
Center of Materials Physics and Chemistry, and Department of Physics, Beihang University, Beijing 100191, People's Republic of China

Dr. G. Casillas
Electron Microscopy Centre, University of Wollongong, Wollongong, NSW 2525, Australia

Prof. W. C. Hao
School of Physics, Beihang Univeristy, Haidian District, Beijing 100091, China

[+] These authors contributed equally to this work.

Keywords: germanene, Dirac fermion, scanning tunneling microscopy, transmission electron microscopy

Supplementary Note 1: STM images of Ag_2Ge alloy.

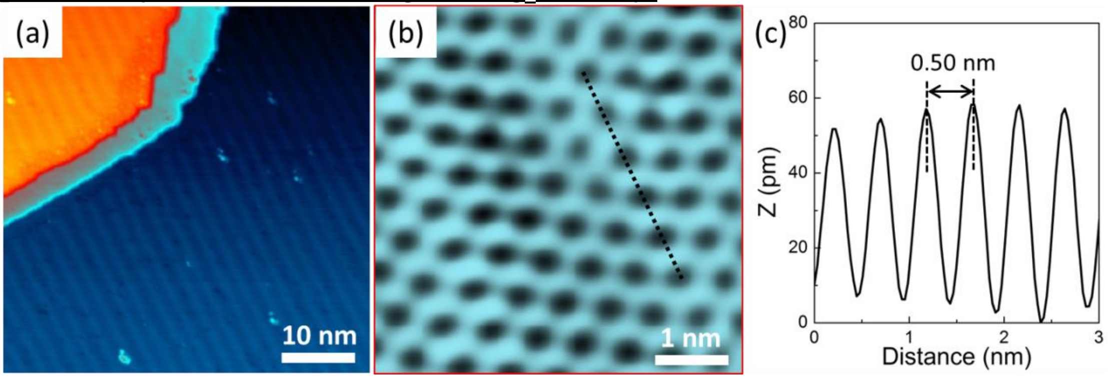

Figure S1. (a) Large-scale STM image of Ag_2Ge alloy. (V_{bias} = -3 V, I = 50 pA) (b) High resolution STM image of Ag_2Ge alloy. (V_{bias} = -7 mV, I = 50 pA) (c) Line profile along the black dashed line in panel (b). The periodicity of the Ag_2Ge alloy is around 0.50 nm, which is √3 times the lattice constant of Ag(111).

The lattice constant of this phase is around √3 times that of the Ag(111) substrate, while the lattice vectors are rotated by 30°. Thus, the structure corresponds to the (√3×√3)R30° superstructure in terms of the 1×1 Ag(111), where one out of three Ag atoms in the surface layer is replaced by a Ge atom. The wavelike striped patterns (Supplementary Fig. 1(a)) are the result of the relaxation of the compression force induced by the difference in the radii between Ge atoms and Ag atoms after Ge insertion. The periodicity of the long-range modulation is 6 times as great as the periodicity of the local √3×√3 honeycomb cell. Therefore, the stripes can be essentially described as a 6√3×√3 reconstruction. The deviation of the atomic arrangement from the ideal √3×√3 periodicity can be identified in the high-resolution STM image (Supplementary Fig. 1(b)), where the honeycomb structure appears to be slightly stretched in the valleys compared to the ridges of the stripes.

Supplementary Note 2: STM images of submonolayer Ge adatoms.

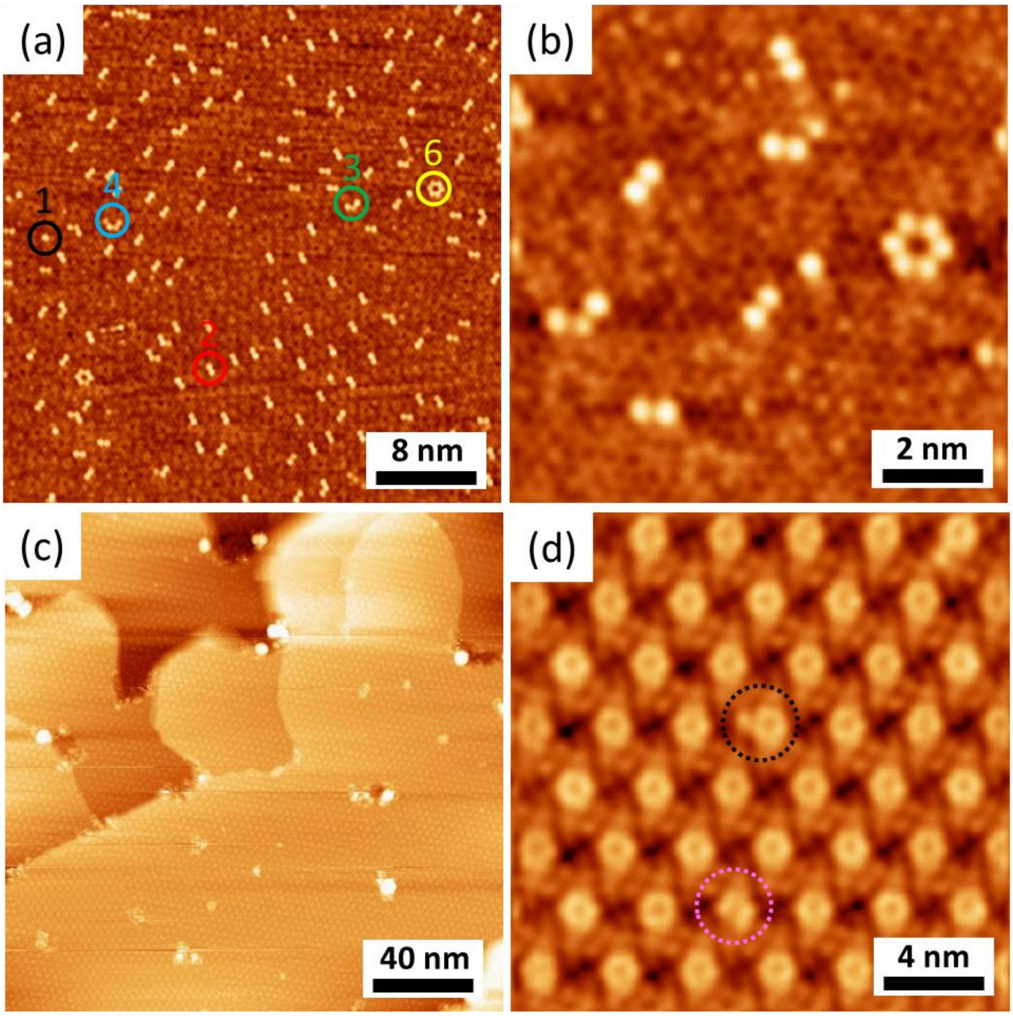

Figure S2. (a) STM image of submonolayer Ge adatoms on the Ag_2Ge alloy surface. (V_{bias} = 0.2 V, I = 50 pA) The black, red, blue, green, and yellow solid rings denote the Ge atoms assembled into a dot, dimer, trimer, tetramer, and hexamer, respectively. (b) Enlarged view of STM image of in panel (a). (V_{bias} = 0.2 V, I = 50 pA) (c) Large-scale STM image of Ge adatoms that have assembled themselves into long-range hexamers. (V_{bias} = 2 V, I = 50 pA) (d) Enlarged view of ordered hexamers. (V_{bias} = 1.5 V, I = 50 pA) The black and pink dashed rings denote a region containing additional adatoms and an area with an incomplete hexamer, respectively.

The structure of Ag_2Ge alloy is destroyed after the additional Ge atoms are deposited (Supplementary Fig. 2). The high resolution STM image (Supplementary Fig. 2b) shows a disordered honeycomb structure with surface adatoms assembling themselves in the forms of dots, dimers, trimers, tetramers, and hexamers. The lattice constant of the disordered honeycomb phase is around 3.92 ± 0.03 Å, which is close to that of simulated free-standing germanene (3.97 - 4.06 Å). Thus, the further deposited Ge atoms “pull out” the Ge atoms in

Ag_2Ge alloy to form the disordered structure due to the stronger interaction between Ge atoms than the interaction between Ag-Ge atoms. Similar results have been reported in **Ref. [1]**, where the phase transition is also correlated to the competitive formation of Ge-Ge covalent and Ge-Ag interfacial bonds. Large-scale ordered hexamers could be observed after more Ge atoms were deposited (Supplementary Fig. 2c), implying that dots, dimers, trimers, and tetramers are the precursor phases of the hexamer. The high resolution STM image (Supplementary Fig. 2d) shows an incomplete hexamer (labelled by the pink dashed ring) and a hexamer with additional adatoms (labelled by the black dashed ring).

Supplementary Note 3: Large-scale STM images of Ge nanosheets.

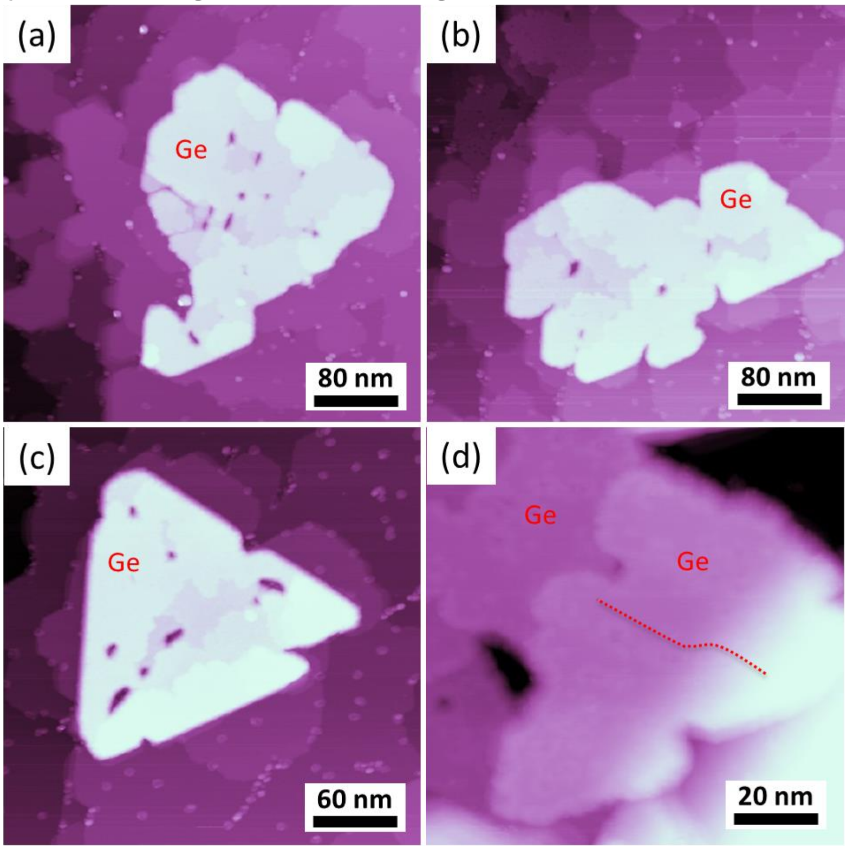

Figure S3. (a)-(c) Large-scale STM image of thick Ge nanosheets with scanning parameters of (V_{bias} = -1.2 V, I = 50 pA), (V_{bias} = 1 V, I = 50 pA), and (V_{bias} = 1 V, I = 50 pA), respectively. (d) STM image of Ge nanosheet bridging two islands with a curly surface curve. (V_{bias} = 2 V, I = 50 pA) The red dashed line is plotted to show the curly surface of Ge nanosheets.

There are some cracks and disconnections in the germanium nanosheets, and some of their sides are in the hexagonal shape (Supplementary Fig. 3a, b, and c). Furthermore, the nanosheets could continuously step over the Ag(111) substrate. Thus, these islands have most likely been assembled from several individual small islands with high mobility on the substrate surface, which manifests the weak interaction between the Ge nanosheets and the Ag(111) substrate. Panel (d) shows a single Ge nanosheet with a curly surface curve bridging two islands regardless of the thickness variation, demonstrating the carpet-mode growth.[2]

Supplementary Note 4: STEM images of Ge nanosheet from cross-sectional view.

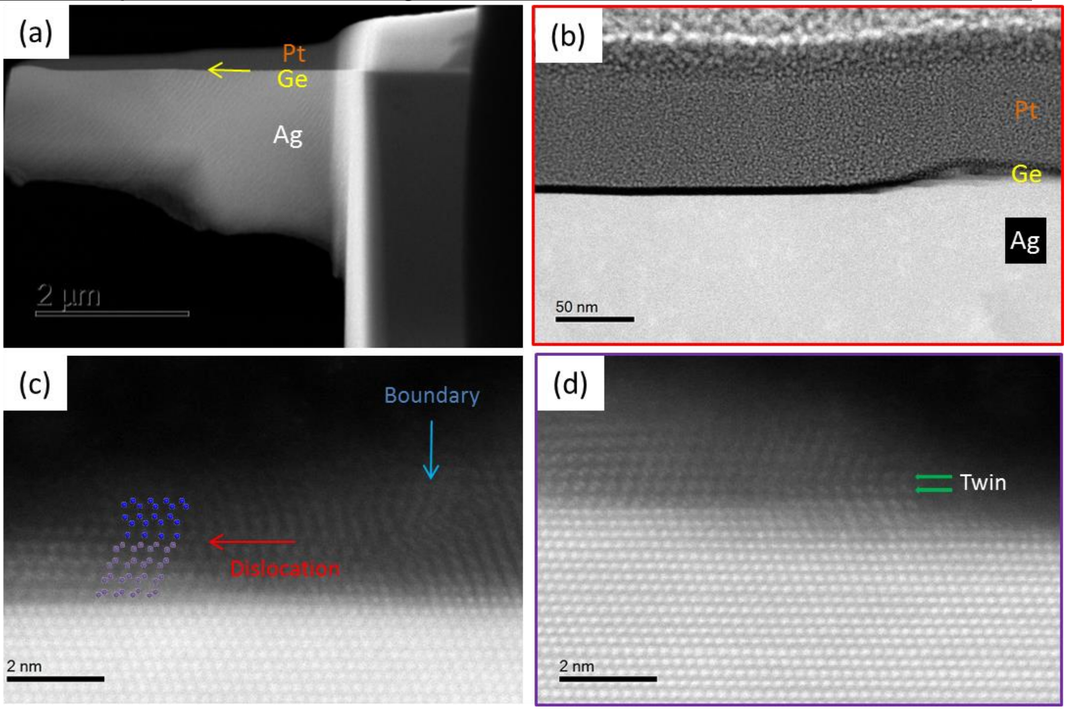

Figure S4. (a) and (b) Wide view, bright field TEM images of the cross-sectional sample, with the protective Pt capping layer, Ge nanosheets, and Ag(111) substrate indicated. (c) and (d) High-resolution AC-STEM images of the FIB sample.

A dislocation and the boundary between the two crystals are labelled in panel (c) by the red arrow and the blue arrow, respectively. Twin crystals with mirror symmetry along the Ag(110) direction are indicated in panel (d). The dislocations, boundaries, and twin crystals are hard to observe in STM images due to the weak dependence of the surface superstructure on the deep crystal structure.

<u>Supplementary Note 5: Raman spectrum of Ag(111) substrate, Ag_2Ge alloy, Ge nanosheets (Ge NS), and bulk Ge(111) crystal.</u>

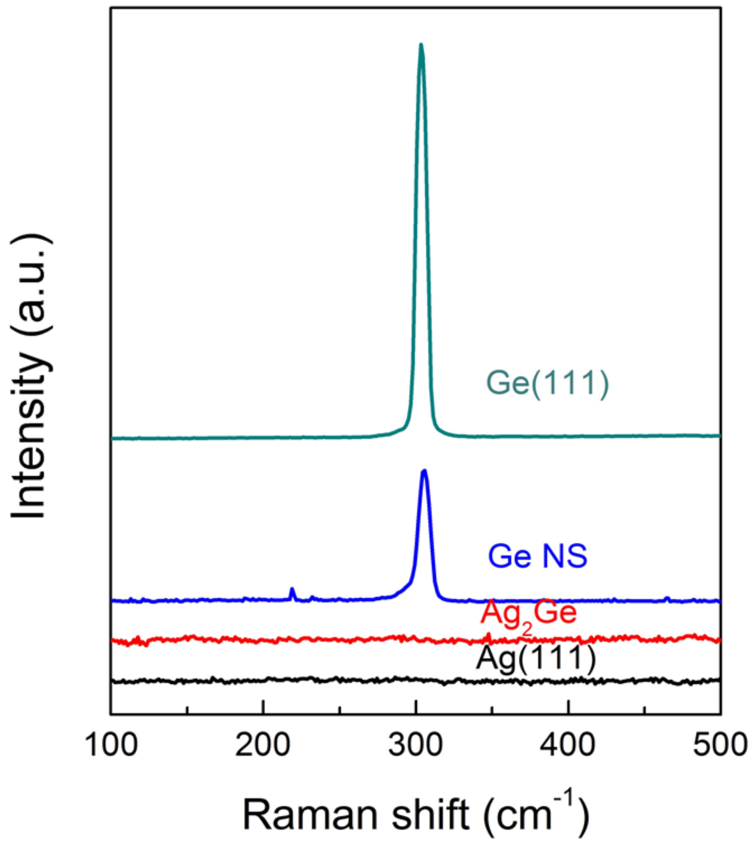

Figure S5. *In-situ* Raman spectrum of Ag(111) substrate, Ag_2Ge alloy, Ge nanosheets (Ge NS), and bulk Ge(111) crystal.

No Raman peaks could be identified for the Ag(111) substrate and the Ag_2Ge alloy because of the Rayleigh scattering and/or the absence of Ge-Ge covalent bonds. For Ge NS and the bulk Ge(111) crystal, the characteristic peak at around 300 cm^{-1} corresponds to the frequency of the in-plane transverse optical (iTO) and in-plane longitudinal optical (iLO) phonon branches at the Γ point for germanium atoms, *i.e.*, a doubly degenerate E_{2g} mode. It is notable that Ge-Ge bond length in the $\sqrt{3}\times\sqrt{3}$ superstructure is similar to that in bulk Ge(111), excluding its strain effect on the shift of the E_{2g} peak, leading to the observation of a single Raman peak in the experimental results.

Supplementary Note 6: Interaction strength between √3×√3 germanene and Ge(111).

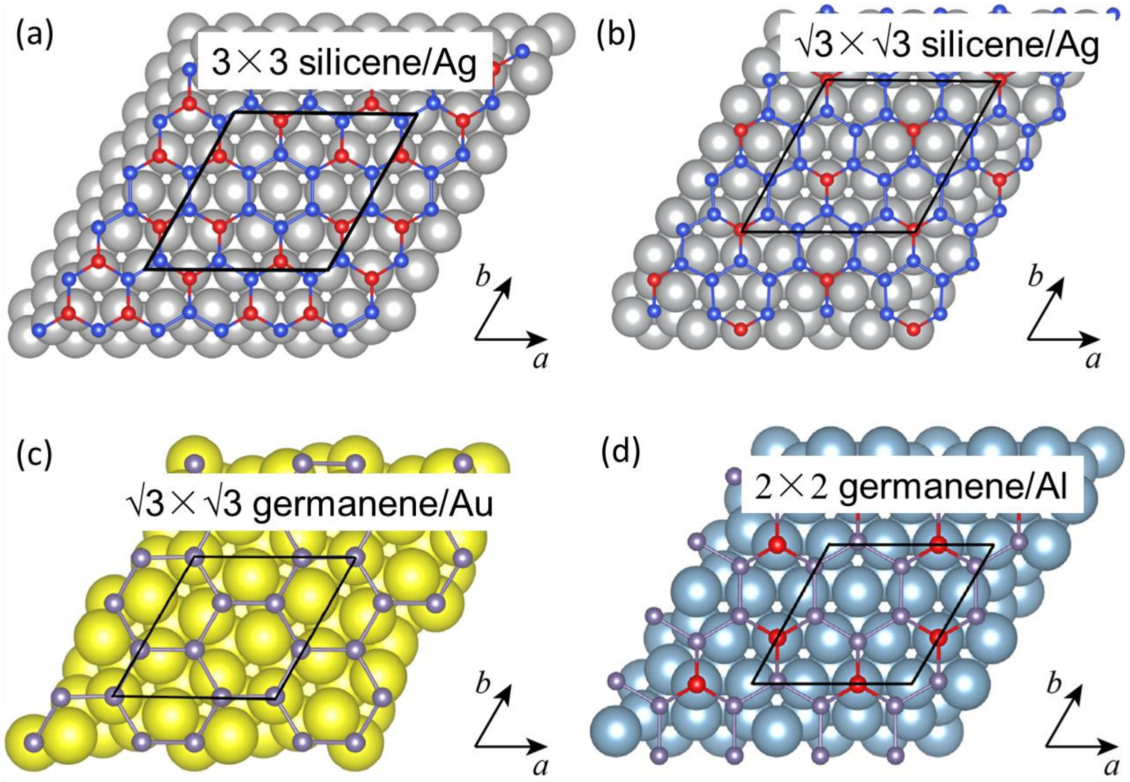

Figure S6. Top view of atomic structures of (a) 3×3 silicene on Ag(111),[4] (b) √3×√3 silicene on Ag(111),[5] (c) √3×√3 germanene on Au(111),[6] and 2×2 germanene on Al(111).[7]

Table S1. The interaction energy of graphene, silicene, and germanene on various substrates. E_{int} is the interaction energy per atom on the topmost layer.

**Model**	E_{int}**/eV**
Graphene on C-terminated SiC(111)[3]	-0.90[3]
Silicene on Ag(111)-I[4]	-0.76
Silicene on Ag(111)-II[5]	-0.68
Germanene on Al(111)[6]	-0.93
Germanene on Au(111)[7]	-0.99
Germanene on Ge(111)*	-0.74

*This wok. Germanene with 4 layer thick Ge(111).

We compared the calculated interaction energy (E_{int}) between √3×√3 germanene and Ge(111) with the interaction energies between graphene/silicene/germanene and their various substrates (Supplementary Fig. 9 and Table 1). The interaction energy per atom of √3×√3

germanene on Ge(111) surfaces is calculated by $E_{int} = (E_{g\text{-}Ge} - E_g - E_{Ge})/N$, where $E_{g\text{-}Ge}$, E_g, and E_{Ge} are total energy of √3×√3 germanene on 4 layer Ge(111) surface after elastic adjustment, free-standing √3×√3 germanene and √3×√3 germanene per unit cell, respectively. N is the number of Ge atoms per unit cell of √3×√3 germanene. It is identified that the value of E_{int} between √3×√3 germanene and Ge(111) essentially locates at the energy range of E_{int} of these well-recognized 2D materials. All these results demonstrate that the interaction between √3×√3 germanene and Ge(111) is weaker than the interlayer interaction in Ge(111), indicating the 2D nature of observed √3×√3 germanene in topmost layer.

Supplementary Note 7: STS maps of germanene and Ag(111) substrate with surface defects.

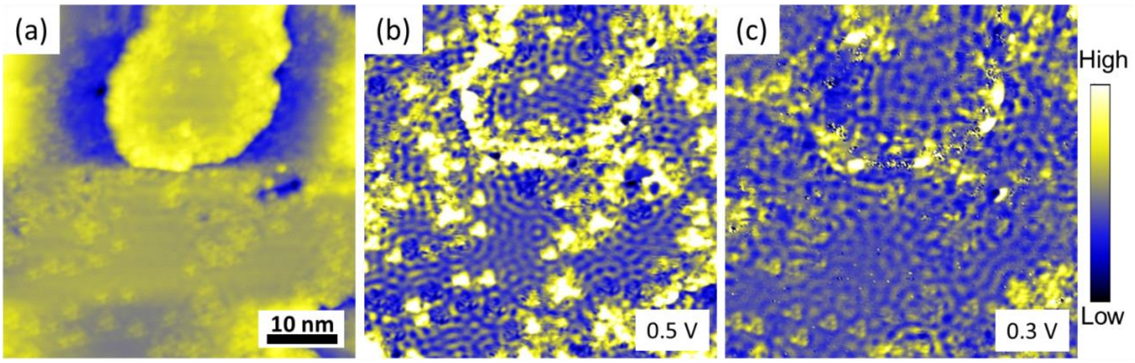

Figure S7. (a) Topography of √3×√3 supercell with point defects and step edges. (V_{bias} = -0.5 V, I = 300 pA) (b) and (c) STS maps of the same area as panel (a), collected with the bias at 0.5 V and 0.3 V, respectively.

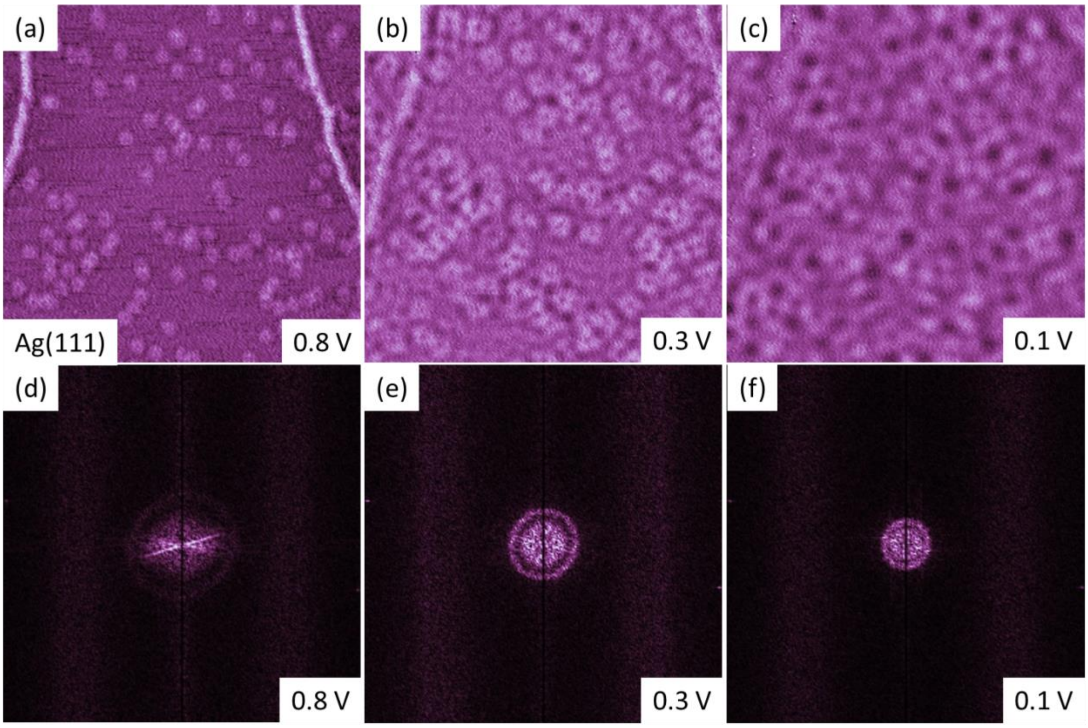

Figure S8. (a)-(c) STS maps of the same area of the Ag(111) surface performed with the bias at 0.8 V, 0.3 V, and 0.1 V, respectively. (40 nm × 40 nm) (d)-(f) k-space maps obtained from the FFT of the STS maps at bias of 0.8 V, 0.3 V, and 0.1 V, respectively. The circular shape regardless of the bias voltage is in good agreement with the typical two-dimensional electron gas (2DEG) case.

Supplementary Note 8: DFT simulations of projected band structures on the topmost germanene for isolated 1–5 layered Ge(111) films

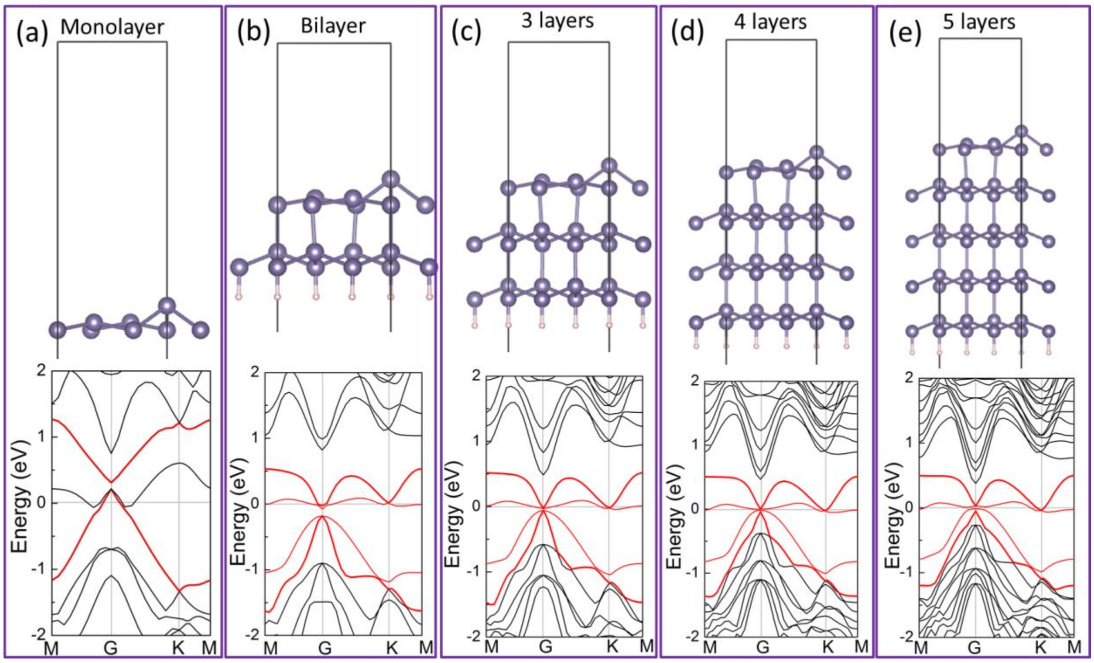

Figure S9. (a)-(e) Side views of monolayer, bilayer, 3-layer, 4-layer, and 5 layer Ge(111) films with the √3×√3 surface structure, and projected band structures on the topmost germanium atomic layers for isolated 1–5 layer Ge(111) films. The red lines denote the Dirac cone bands.

Supplementary Note 9: Numerical results on electronic band structures in √3×√3 germanene.

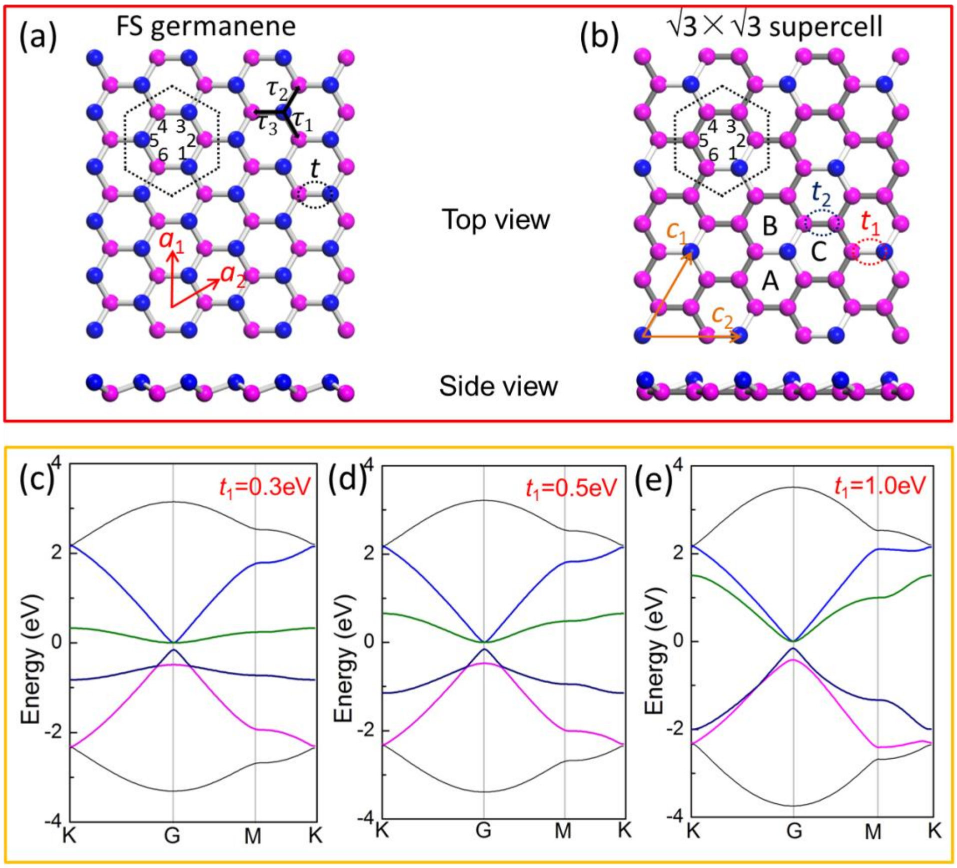

Figure S10. (a) and (b) Schematic diagrams of the lattice structure of FS germanene and the √3×√3 superstructure from both the top view and the side view, respectively. ($\mathbf{a}_1$, $\mathbf{a}_2$) and ($\mathbf{c}_1$, $\mathbf{c}_2$) represent the lattice vectors of FS germanene and the √3×√3 restructured germanene, respectively. In FS germanene, all the hopping amplitudes have the same value, t. Two types of Ge-Ge bonds in the √3×√3 supercell, denoted as t_1 and t_2, are distinguished due to the presence of one buckled-up atom in the unit cell (six Ge atoms). (c)-(e) Numerical results on the electronic structure of √3×√3 germanene with varied hopping parameter t_1.

Germanium atoms on sublattices A and B are depicted by filled (*e.g.* Nos. 1, 3, 5 in the dashed hexagon in Supplementary Fig. 13a) and open black circles (*e.g.* Nos. 2, 4, 6 in the dashed hexagon in Supplementary Fig. 13a). The vectors τ point from one Ge atom on sublattice A to its three nearest neighbors on sublattice B. Two hopping parameters t_1 and t_2 are marked in Supplementary Fig. 13b.

First, if the differences in the on-site energies are ignored, the Hamiltonian of the superstructure could be written as:

$$H(k)=-\begin{pmatrix} 0 & t_1e^{i\vec{k}.\tau_3} & 0 & t_1e^{i\vec{k}.\tau_1} & 0 & t_1e^{i\vec{k}.\tau_2} \\ t_1e^{-i\vec{k}.\tau_3} & 0 & t_2e^{-i\vec{k}.\tau_1} & 0 & t_2e^{-i\vec{k}.\tau_2} & 0 \\ 0 & t_2e^{i\vec{k}.\tau_1} & 0 & t_2e^{i\vec{k}.\tau_2} & 0 & t_2e^{i\vec{k}.\tau_3} \\ t_1e^{-i\vec{k}.\tau_1} & 0 & t_2e^{-i\vec{k}.\tau_2} & 0 & t_2e^{-i\vec{k}.\tau_3} & 0 \\ 0 & t_2e^{i\vec{k}.\tau_2} & 0 & t_2e^{i\vec{k}.\tau_1} & 0 & t_2e^{i\vec{k}.\tau_3} \\ t_1e^{-i\vec{k}.\tau_2} & 0 & t_2e^{-i\vec{k}.\tau_1} & 0 & t_2e^{-i\vec{k}.\tau_3} & 0 \end{pmatrix}$$

And the characteristic equation is：

$$E^6-3E^4t_1^2-6E^4t_2^2-4E^2t_1^2t_2^2\left[\cos\left(3\vec{k}.\vec{\tau}_1\right)+\cos\left(3\vec{k}.\vec{\tau}_2\right)+\cos\left(3\vec{k}.\vec{\tau}_3\right)-3\right]+2E^2t_2^4\left[\cos\left(3\vec{k}.\vec{\tau}_1\right)+\cos\left(3\vec{k}.\vec{\tau}_2\right)+\cos\left(3\vec{k}.\vec{\tau}_3\right)-3\right]$$
$$-2t_1^2t_2^4\left\{3\cos\left(3\vec{k}.\vec{\tau}_1\right)+3\cos\left(3\vec{k}.\vec{\tau}_2\right)+3\cos\left(3\vec{k}.\vec{\tau}_3\right)-6-\cos\left[3\vec{k}(\vec{\tau}_2-\vec{\tau}_3)\right]-\cos\left[3\vec{k}(\vec{\tau}_1-\vec{\tau}_3)\right]-\cos\left[3\vec{k}(\vec{\tau}_1-\vec{\tau}_2)\right]\right\}=0$$

When $k = 0$ (at the Γ point), it can be simplified to:

$$E^6-3E^4t_1^2-6E^4t_2^2=E^4(E^2-3t_1^2-6t_2^2)=0$$

Therefore, there will be four degenerate roots with zero values, implying that there would be no open gap at the Γ point of the Brillouin zone of the superstructure.

Second, from our DFT calculations, the on-site energies are different in the superstructure. The Hamiltonian of the superstructure could be written as:

$$H(k)=-\begin{pmatrix} -\varepsilon_1 & t_1e^{i\vec{k}.\tau_3} & 0 & t_1e^{i\vec{k}.\tau_1} & 0 & t_1e^{i\vec{k}.\tau_2} \\ t_1e^{-i\vec{k}.\tau_3} & -\varepsilon_3 & t_2e^{-i\vec{k}.\tau_1} & 0 & t_2e^{-i\vec{k}.\tau_2} & 0 \\ 0 & t_2e^{i\vec{k}.\tau_1} & -\varepsilon_2 & t_2e^{i\vec{k}.\tau_2} & 0 & t_2e^{i\vec{k}.\tau_3} \\ t_1e^{-i\vec{k}.\tau_1} & 0 & t_2e^{-i\vec{k}.\tau_2} & -\varepsilon_3 & t_2e^{-i\vec{k}.\tau_3} & 0 \\ 0 & t_2e^{i\vec{k}.\tau_2} & 0 & t_2e^{i\vec{k}.\tau_1} & -\varepsilon_2 & t_2e^{i\vec{k}.\tau_3} \\ t_1e^{-i\vec{k}.\tau_2} & 0 & t_2e^{-i\vec{k}.\tau_1} & 0 & t_2e^{-i\vec{k}.\tau_3} & -\varepsilon_3 \end{pmatrix}$$

In the current model, we set the values of the on-site energies based on the DFT calculations, where ε_1 = 0.50 eV, ε_2 = 0.15 eV, and ε_3 = 0.00 eV. When t_2 is determined by the DFT band structure, the dispersion relation can be achieved by tuning t_1 (see detailed discussion on calculation in Main text). It was found that the two bands close to zero energy become more flat as the value of t_1 decreases (Supplementary Fig. 13). The band structure around the Γ point is similar to the DFT result (Fig. 4f) with t_1 = 0.30 eV. The numerical results agree well with the physical picture, that forming the superstructure results in decreasing orbital overlapping and increasing orbital energy for the buckled-up Ge atoms, which means a smaller t_1 and larger ε_1 in the numerical model, respectively. It reveals the origin of opened energy gap in germanene supported by Ge(111) film. The features far from Γ point are not close to the DFT band is due to the less orbitals compared to these included in the calculations.

[1] C. H. Lin, A. Huang, W. W. Pai, W. C. Chen, T. Y. Chen, T. R. Chang, R. Yukawa, C. M. Cheng, C. Y. Mou, I. Matsuda, T. C. Chiang, H. T. Jeng, S. J. Tang, *Phys. Rev. Mater.* **2018**, *2*, 024003.

[2] H. I. Rasool, E. B. Song, M. Mecklenburg, B. C. Regan, K. L. Wang, B. H. Weiller, J. K. Gimzewski, *J. Am. Chem. Soc.* **2011**, *133*, 12536.

[3] X. Shan, Q. Wang, X. Bian, W. Li, G. Chen, H. Zhu, *RSC Adv.* **2015**, *5*, 78625.

[4] P.Vogt, P. De Padova, C. Quaresima, J. Avila, E. Frantzeskakis, M. C. Asensio, A. Resta, B. Ealet, G. Le Lay, *Phys. Rev. Lett.* **2012**, *108*, 155501.

[5] L. Chen, H. Li, B. Feng, Z. Ding, J. Qiu, P. Cheng, K. Wu, S. Meng, *Phys. Rev. Lett.* **2013**, *110*, 085504

[6] M. Derivaz, D. Dentel, R. Stephan, M. C. Hanf, A. Mehdaoui, P. Sonnet, C. Pirri, *Nano Lett.* **2015**, *15*, 2510.

[7] M. E. Dávila, L. Xian, S. Cahangirov, A. Rubio, G. Le Lay, *New J. Phys.* **2014**, *16*, 095002.
